# PPARγ-independent induction of growth arrest and apoptosis in prostate and bladder carcinoma

**DOI:** 10.1186/1471-2407-6-53

**Published:** 2006-03-06

**Authors:** Christine L Chaffer, David M Thomas, Erik W Thompson, Elizabeth D Williams

**Affiliations:** 1 Bernard O'Brien Institute of Microsurgery, Victoria, Australia; 2 Ian Potter Foundation Centre for Cancer Genomics and Predictive Medicine, Peter MacCallum Cancer Centre, Victoria, Australia; 3 The University of Melbourne Department of Surgery, St. Vincent's Hospital, Victoria, Australia; 4 St Vincent's Institute of Medical Research, Victoria, Australia

## Abstract

**Background:**

Although PPARγ antagonists have shown considerable pre-clinical efficacy, recent studies suggest PPARγ ligands induce PPARγ-independent effects. There is a need to better define such effects to permit rational utilization of these agents.

**Methods:**

We have studied the effects of a range of endogenous and synthetic PPARγ ligands on proliferation, growth arrest (FACS analysis) and apoptosis (caspase-3/7 activation and DNA fragmentation) in multiple prostate carcinoma cell lines (DU145, PC-3 and LNCaP) and in a series of cell lines modelling metastatic transitional cell carcinoma of the bladder (TSU-Pr1, TSU-Pr1-B1 and TSU-Pr1-B2).

**Results:**

15-deoxy-prostaglandin J_2 _(15dPGJ2), troglitazone (TGZ) and to a lesser extent ciglitazone exhibited inhibitory effects on cell number; the selective PPARγ antagonist GW9662 did not reverse these effects. Rosiglitazone and pioglitazone had no effect on proliferation. In addition, TGZ induced G0/G1 growth arrest whilst 15dPGJ2 induced apoptosis.

**Conclusion:**

Troglitazone and 15dPGJ2 inhibit growth of prostate and bladder carcinoma cell lines through different mechanisms and the effects of both agents are PPARγ-independent.

## Background

The transcription factor peroxisome proliferator-activated receptor gamma (PPARγ) has sparked significant interest in the cancer field owing to observations of increased expression in several diverse carcinomas [1-4]. Moreover, endogenous and synthetic PPARγ agonists elicit notable growth inhibitory effects *in vitro *and *in vivo *(colon, breast and prostate carcinomas) and are capable of preventing metastasis [2,5-9]. PPARγ is a member of the family of nuclear hormone receptors and exists as two isoforms (PPARγ1 and PPARγ2) encoded by multiple transcript variants [10]. PPARγ1 is the predominant isoform in humans and is highly expressed in adipose tissue and liver [8,10]. Historically, PPARγ is recognized as a critical transcription factor in the regulation of adipocyte differentiation and genes involved in energy storage and utilisation [11], and PPARγ agonists (rosiglitazone, pioglitazone) are currently in clinical use for the treatment of Type II diabetes [12].

Several high affinity ligands are routinely used to investigate PPARγ-mediated effects, including the family of synthetic thiazolidinedione ligands (rosiglitazone, RGZ; pioglitazone, PGZ; ciglitazone, CGZ; troglitazone, TGZ; listed from highest to lowest affinity (K_d _30–750 nM) for PPARγ) and the endogenous ligand 15-deoxy-prostaglandin J_2 _(15dPGJ2), a metabolite of prostaglandin D_2 _(K_d_~300 nM) [13,14]. While previous studies demonstrate that some PPARγ ligands inhibit growth of multiple carcinoma cell lines [6,15-17], many reports demonstrate that PPARγ ligand-mediated growth inhibition can vary depending on the cancer type. In astroglioma, colorectal, and hepatocellular carcinoma, as well as in astrocytes and preadipocytes, growth inhibition can be limited to a particular member of the thiazolidinedione family [18-21], and has been shown to be selective for the endogenous PPARγ ligand, 15dPGJ2, over members of the thiazolidinedione family [22]. Indeed, even within a carcinoma type, growth inhibition induced by PPARγ ligands can be cell line specific [23]. In addition, studies utilising the irreversible PPARγ-selective antagonist GW9662, have revealed PPARγ-dependent and -independent mechanisms of growth inhibition [21,24-26], further highlighting the incongruity of responsiveness between cancer types.

The high incidence of prostate and bladder carcinoma poses a significant health risk for men, including Australian and North American males Interestingly, the expression of PPARγ is increased with grade and advancement of disease [1,3]. In clinical trials of patients with advanced prostate cancer, treatment with TGZ has resulted in prolonged stabilisation of prostate specific antigen (PSA) levels [28,29]. Although genetic deficiency of PPARγ does not alter the development of experimental prostate cancer [30], individual PPARγ ligands have been shown to inhibit *in vitro *cellular proliferation of both human bladder and prostate carcinoma cell lines [1,2,31-34]. In prostate carcinoma cell lines the antiproliferative effects of these agonists have also been associated with morphological changes indicative of terminal differentiation [34] and a less malignant phenotype [2].

The antineoplastic properties of the thiazolidinedione ligands have offered promise in clinical and pre-clinical studies of prostate carcinoma, but are yet to be fully characterised in bladder carcinoma. In other cancer types, it is becoming evident that growth inhibition can be ligand dependent, and cannot always be attributed to PPARγ activation. These studies also illustrate that the specific PPARγ agonist utilised critically determines the outcome. In this study we have utilized a broad range of PPARγ agonists and the PPARγ-selective antagonist GW9662, to examine the effect of PPARγ activation in prostate and bladder carcinoma cell lines.

## Methods

### Cell lines and reagents

TSU-Pr1, DU145, PC-3 and LNCaP cell lines were obtained from Dr Dan Djakiew, Georgetown University, USA [35]; TSU-Pr1-B1 and TSU-Pr1-B2 showing increasing metastatic potential were generated in our laboratory by *in vivo *selection to bone ; HL60 cell line was obtained from the Peter McCallum Cancer Centre, Australia; 3T3-L1 cell line was obtained from the American Type Culture Collection (Rockville, MD). All cell lines were cultured in DMEM (Gibco, Australia) supplemented with 10% foetal bovine serum (FBS, JRH Biosciences, Australia). Rosiglitazone (RGZ), troglitazone (TGZ), pioglitazone (PGZ), ciglitazone (CGZ), 15dPGJ2 and GW9662 were purchased from Sapphire Biosciences (Australia) and were dissolved in dimethylsulfoxide (DMSO) (Sigma, Australia). Doxorubicin (Dox) and Sulforhodamine B (SRB) were purchased from Sigma.

### qRT-PCR

Total RNA was isolated using RNeasy Mini Kit (Qiagen, Australia) as per the manufacturer's instructions. RNA was reverse transcribed (Superscript II; Invitrogen, Australia) to cDNA using random hexamers (Invitrogen, Australia). Quantitative RT-PCR was performed on an ABI Prism 5700 Sequence Detection System (Perkin-Elmer Applied Biosystems; Australia) in 10 mM Tris-HCl pH 8.0, 2.5 mM Mg(C_2_H_3_O_2_)_2_, 50 mM KCl, 200 μM dNTPs, 1/40,000 dilution of SYBR Green I (Molecular Probes; Eugene, Oregon USA), 1 μg/ml 6-carboxy-X-rhodamine (6-ROX; Molecular Probes), 8% DMSO, 200 nM primers and 0.625 U AmpliTaq Gold polymerase (Applied Biosystems) per 25 μL reaction. Levels of PPARγ1,3 transcript variants (NM_138712 and NM_138711; primers: 5'-CCATTTTCTCAAACGAGAGTCAGCCTTT-3' and 5'- CTCTGTGTCAACCATGGTCATTTCGTT-3'), PPARγ2 transcript variant (NM_015869 ; primers: 5'-GATGTCTTGACTCATGGGTGTATTCACAAA-3' and 5'- GTTTGCAGACAGTGTATCAGTGAAGGAA-3') and PPARγ4 transcript variant (NM_005037; primers: 5'-CGCCGTGGCCGCAGAAAT-3' and 5'- GATCCACGGAGCTGATCCCAAA-3') were measured. Each primer set gave rise to predicted sized amplicons of 55, 202 and 80 bp respectively. L32 (NM_000994; primers: 5'-CAGGGTTCGTAGAAGATTCAAGGG-3' and 5'-CTTGGAGGAAACATTGTGAGCGATC-3'; amplicon: 190 bp), a ribosome associated protein, was used as a housekeeping gene to normalise all samples as previously described [36]. All reactions were performed in quadruplicate. PCR conditions were 95°C, 10 min followed by 50 cycles of reaction at 95°C, 15 s, 60°C, 1 min. Specificity of PCR products was determined by dissociation curve analysis as recommended by the manufacturer (ABI) and products were confirmed to be single bands by visualisation following gel electrophoresis. The comparative CT method was validated by assessing the amplification efficiency of each of the primer sets listed above in a single PCR run as per the manufacturer's recommendations (ABI).

### Proliferation analysis

Cell number was estimated using the SRB assay. Cells were seeded in 96-well plates at a starting density of 1 × 103 cells/well and treated with PPARγ ligand or control vehicle (0.2 % DMSO) in the presence or absence of GW9662 (10 μM) the following day. At days 0, 1, 3, 5, 7 and 9, cells were fixed with 25 μL cold (4°C) 50% trichloroacetic acid for 1 hr at 4°C. Cells were stained with 100 μL SRB (0.4 g/100 mL in 1% acetic acid) for 15 minutes at room temperature (RT), washed with 1% acetic acid and left to dry overnight. Bound SRB was resuspended in 10 mM Tris base (pH 10.5) and read at 550 nm on a Labsystems Multiskan RC spectrophotometer.

### Cell cycle analysis

Cells were seeded in six 10 cm dishes at varying densities according to ligand treatment (1 × 105 cells/plate; control, RGZ 10 μM, TGZ 50 μM and 15dPGJ2 5.6 μM and 3 × 105 cells/plate; TGZ 100 μM and 15dPGJ2 10 μM) and after 24 hrs media was replaced with fresh media containing the specific PPARγ ligand treatment or control vehicle (0.1% DMSO) for 72 hrs. Floating and adherent cells were combined, pelleted (400 g), washed in phosphate buffered saline (PBS) and resuspended in 200 μL PBS containing 0.1% FBS. The cell suspension was diluted 10-fold in ice-cold 90% ethanol and placed at -20°C for 15 minutes. Cells were then washed twice with 0.1% FBS in PBS and resuspended in RNase (2 μg/mL) (Roche, Australia) and 5 mg/mL Propidium Iodide (Sigma). Flow cytometric analysis of DNA content was performed using a FACScalibar (Becton Dickinson) with Cellquest software (Becton Dickinson) and analysed using Modfit cell cycle analysis software (Verity Software House, Maine) as previously described

### Determination of DNA fragmentation

DNA was extracted using the Apoptotic DNA Ladder Kit (Roche), according to the manufacturer's instructions. Briefly, cells were seeded in 10 cm dishes and after 24 hrs media was replaced with fresh media containing the specific PPARγ ligand, doxorubicin (Dox, 1 μM) or control vehicle (0.1% DMSO) for 72 hrs. Floating and adherent cells were combined, lysed, applied to a column, and the DNA eluted. Samples were treated with RNase (2 μg/mL, 15 mins, RT). DNA was run on a 1% agarose gel containing ethidium bromide and visualised on a UV transilluminator (Promega, Australia).

### Caspase activation

Levels of active caspase-3/7 after ligand treatment were measured using the CaspaTag™ Caspase-3/7 *In situ *Assay Kit (Chemicon International, Australia), according to the manufacturer's instructions. Briefly, 1 × 10^6^cells (Control, RGZ 10 μM, TGZ 50 μM and 15dPGJ2 5.6 μM) and 3 × 10^6^cells (TGZ 100 μM, 15dPGJ2 10 μM, Dox 1 μM) were seeded in 15 cm dishes and treated for 48 hrs to induce apoptosis. Cells were then exposed to a carboxyfluorescein-labeled fluoromethyl ketone inhibitor of caspase-3/7 (FAM-DEVD-FMK) and transferred to a microtiter plate. Levels of active caspase-3/7 were measured by reading the fluorescence of each microwell with excitation (485 nm) and emission (535 nm) wavelengths using a Wallac 1200 spectrophotometer.

### Statistical analysis

Results are expressed as mean ± standard error (SEM) from 2–3 experiments performed in triplicate. Unpaired t test or ANOVA followed by the Dunnett post test were used to determine statistical significance. *P *< 0.05 was considered significant.

## Results

### Levels of PPARγ mRNA in bladder and prostate cell lines

**Figure 1:**
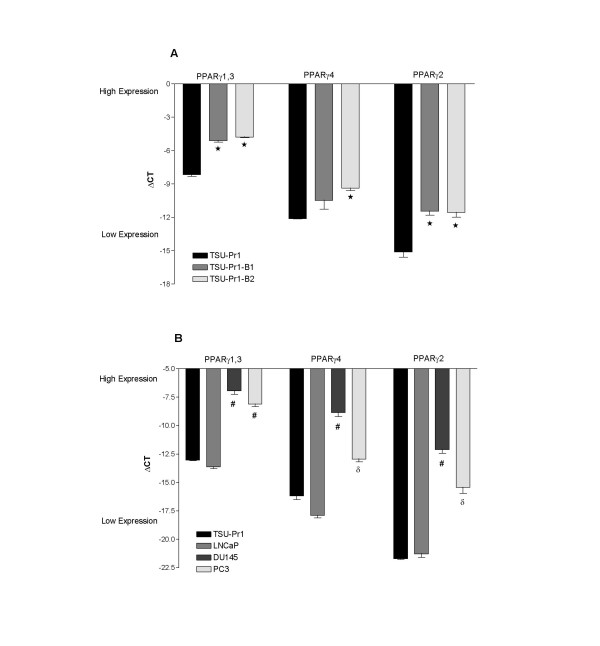
**Detection of PPARγ isoforms in bladder (TSU-Pr1, TSU-Pr1-B1, TSU-Pr1-B2) and prostate (DU145, PC-3 and LNCaP) cell lines**. PPARγ1 (encoded by PPARγ variants 1, 3 and 4) and PPARγ2 isoforms were measured by qRT-PCR. ΔCT values were derived by normalisation to levels of the L32 housekeeping gene in each cDNA sample. * Significantly different to TSU-Pr1 (p < 0.001), ^# ^significantly different to TSU-Pr1 and LNCaP, (p < 0.01), ^δ ^significantly different to DU145 (p < 0.03) (unpaired t test)

We examined the expression of PPARγ in TSU-Pr1 and sublines with increasing metastatic potential (Figure 1A). PPARγ1,3, PPARγ2 and PPARγ4 transcript variants were detected in all cell lines. PPARγ1,3 was expressed at the highest levels in all three cell lines, followed by PPARγ4 (these transcript variants detect PPARγ isoform 1). PPARγ2 (PPARγ isoform 2) was expressed at the lowest level in all three cell lines. Interestingly, the levels of both PPARγ isoforms were significantly up-regulated in the more metastatic sublines TSU-Pr1-B1 and TSU-Pr1-B2 compared to TSU-Pr1 (with the exception of the PPARγ4 levels in TSU-Pr1-B1 compared to TSU-Pr1).

The prostate cell lines DU145, PC-3 and LNCaP were also examined for PPARγ expression and compared to expression levels in TSU-Pr1 (Figure 1B). The levels of all transcript variants were similar in TSU-Pr1 and LNCaP cell lines with PPARγ1,3 expressed at the highest level and PPARγ2 at the lowest level. All transcript variants were expressed at significantly higher levels in DU145 and PC-3 cell lines compared to TSU-Pr1 and LNCaP cell lines. In addition, the expression level of PPARγ4 and PPARγ2 were significantly higher in DU145 cells compared to the PC-3 cell line.

### Thiazolidinedione ligands and 15dPGJ2 decrease cell number in bladder cell line TSU-Pr1

**Figure 2:**
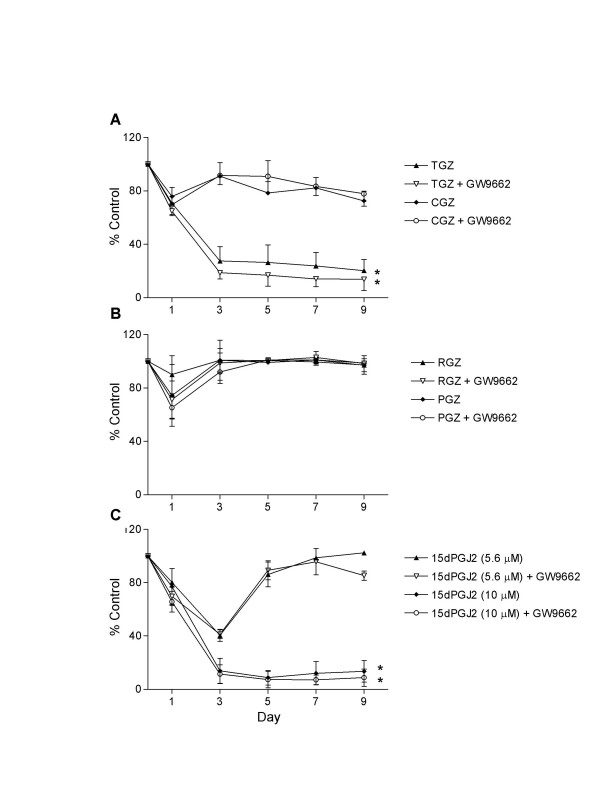
**Effect of PPARγ ligands on TSU-Pr1 cell number**. Cells were treated with ligand either alone or in the presence of 10 μM GW9662 at Day 0. *A*, Troglitazone (100 μM; TGZ) and ciglitazone (40 μM; CGZ). *B*, Rosiglitazone (10 μM; RGZ) and pioglitazone (20 μM; PGZ). *C*, 15-deoxy-prostaglandin J_2 _(15dPGJ2) 5.6 μM and 10 μM. Values are mean ± SEM of 2–3 experiments performed in triplicate expressed as percent control. Statistical significance * P < 0.01 (ANOVA followed by Dunnett post test).

The effects of TGZ (50 and 100 μM), CGZ (40 μM), RGZ (10 μM), PGZ (20 μM) and 15dPGJ2 (5.6 and 10 μM) on the TSU-Pr1 bladder carcinoma cell line were examined in the presence or absence of the highly selective PPARγ antagonist GW9662 (10 μM). This concentration of GW9662 inhibited TGZ- and RGZ-induced lipid accumulation in differentiating NIH3T3-L1 cells (data not shown). The effects of ligand treatment on cell number were determined at days 0, 1, 3, 5, 7 and 9. TGZ (100 μM) induced a marked inhibition of cell number (75% inhibition at day 3), which was sustained throughout the time course of the experiment (Figure 2A). TGZ had no effect on cell number at a lower dose (50 μM) (data not shown). CGZ caused a small decrease (approximately 25%) in cell number at day 1, which subsided to a modest 10–15 % inhibition over days 3 to 9 (Figure 2A). The more potent PPARγ ligands RGZ and PGZ did not affect TSU-Pr1 cell number (Figure 2B). The endogenous PPARγ ligand 15dPGJ2 exhibited a potent and sustained effect on cell number at 10 μM (92% inhibition at day 3). At a lower dose (5.6 μM), 15dPGJ2 initially inhibited cell number (60% at day 3), however this effect was no longer evident by day 5 (Figure 2). It is possible that 15dPGJ2 is either breaking down or being metabolised such that the 5.6 μM treatment is unable to maintain suppression of proliferation over the course of the experiment. The growth inhibitory effects of TGZ, CGZ and 15dPGJ2 on cell number were not reversed by 10 μM GW9662 (Figure 2A–C). Furthermore, at doses ranging from 1 to 100 μM, GW9662 had no effect on TGZ-induced growth inhibition of TSU-Pr1 cells (data not shown).

### Thiazolidinedione ligands and 15dPGJ2 decrease cell number in TSU-Pr1-B1 and TSU-Pr1-B2 sublines

**Figure 3:**
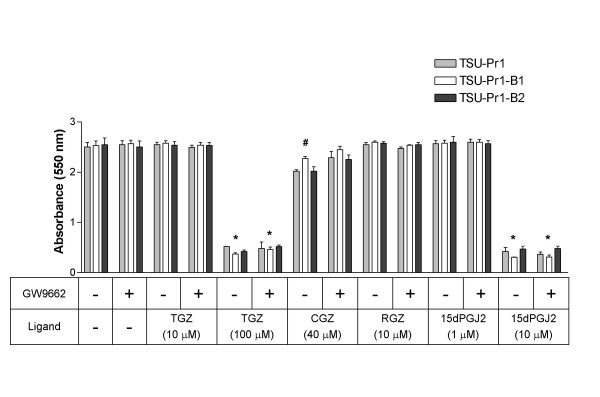
**Effect of PPARγ ligands on cell number of TSU-Pr1/TSU-Pr1-B1/TSU-Pr1-B2 series day 5**. TSU-Pr1, TSU-Pr1-B1 and TSU-Pr1-B2 cells were treated with control (0.2% DMSO), GW9662 (10 μM), troglitazone (10 and 100 μM; TGZ), ciglitazone (40 μM; CGZ), rosiglitazone (10 μM; RGZ) and 15-deoxy-prostaglandin J2 (1 and 10 μM; 15dPGJ2) in the presence or absence of antagonist GW9662 (10 μM) at day 0. Values are mean ± SEM from Day 5. * All cell lines significantly different to the respective controls, ^# ^TSU-Pr1 and TSU-Pr1-B2 significantly different to the respective controls (unpaired t test).

The effects of thiazolidinedione agonists, 15dPGJ2 and GW9662 were examined in the TSU-Pr1 sublines that have significantly increased expression of PPARγ. TGZ (10 and 100 μM), CGZ (40 μM), RGZ (10 μM), 15dPGJ2 (1 and 10 μM) were applied to the cells in the presence or absence of GW9662 (10 μM). Cell number was determined over a 9 day period and the results from Day 5 have been represented (Figure 3). As was observed in TSU-Pr1 cells (Figure 2 and Figure 3), TGZ (100 μM) and 15dPGJ2 (10 μM) dramatically inhibited growth in TSU-Pr1-B1 and TSU-Pr1-B2 cells. There was no difference in the extent of growth inhibition caused by these ligands between the three cell lines despite varying PPARγ expression levels. Again these effects were not reversed by GW9662.

### Thiazolidinedione ligands and 15dPGJ2 decrease cell number in prostate cell lines

**Figure 4:**
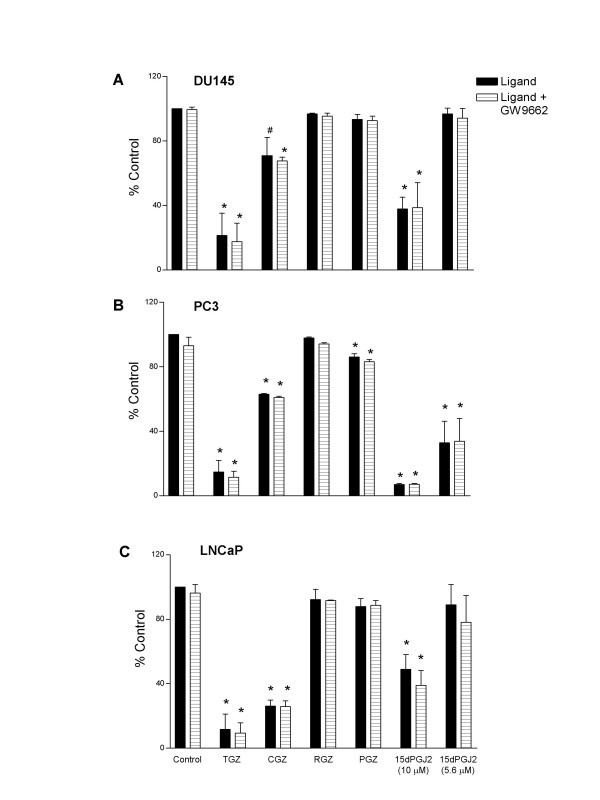
**Effect of PPARγ ligands on cell number of prostate cell lines at day 5**. Cells were treated with control (0.2% DMSO), troglitazone (100 μM; TGZ), ciglitazone (40 μM; CGZ), rosiglitazone (10 μM; RGZ), pioglitazone (20 μM; PGZ) and 15-deoxy-prostaglandin J2 (5.6 and 10 μM; 15dPGJ2) in the presence or absence of antagonist GW9662 (10 μM) at day 0. *A*, DU145 cells; B, PC-3 cells; C, LNCaP cells Values are mean ± SEM of two experiments performed in triplicate expressed as percent control. No significant differences were detected following GW9662 treatment for any agent. Statistical significance compared to control * *P *< 0.01, ^#^*P *< 0.05 (ANOVA followed by Dunnett post test).

The effects of thiazolidinedione agonists, 15dPGJ2 and PPARγ antagonist GW9662 were further examined in a series of prostate cell lines (DU145, PC-3 and LNCaP). LNCaP cells were plated at a higher density (3 × 10^3 ^cells/well) due to their slower mitotic rate. The effects of these ligands have been shown at day 5, when maximal inhibition was observed (Figure 4). Patterns of growth inhibition similar to those seen in TSU-Pr1 cells (Figure 2 and Figure 3) were observed in all prostate carcinoma cell lines. TGZ (100 μM) and 15dPGJ2 (10 μM) treatment had a marked inhibitory effect on cell number (Figure 4A–C). PC-3 cells were more sensitive to the 15dPGJ2 treatment as evidenced by a significant inhibition at the lower dose of 15dPGJ2 (5.6 μM) (Figure 4B), an effect which was not observed in either DU145 (Figure 4A) or LNCaP cell lines (Figure 4C) and only partially seen with TSU-Pr1 (Figure 2 and Figure 3). All prostate carcinoma cell lines showed a significant inhibition of cell number in response to CGZ treatment at day 5, an effect that was most pronounced in LNCaP cells (Figure 4) whereas only a slight effect of CGZ was noted in TSU-Pr1 cells (Figure 2 and Figure 3). As observed in TSU-Pr1 cells, the inhibitory effects exerted by these agonists were not reversed by the addition of the PPARγ antagonist GW9662 (Figure 4) and the thiazolidinedione compounds with the highest affinity for PPARγ (RGZ and PGZ) showed no effect (data not shown).

### TGZ and 15dPGJ2 exert differential effects on cell cycle and apoptosis

**Figure 5:**
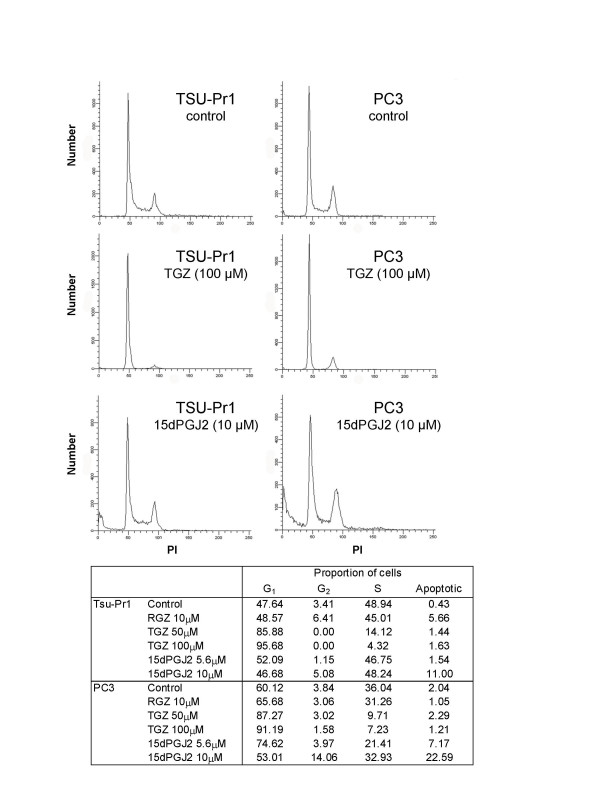
**Effect of PPARγ ligands on cell cycle in TSU-Pr1 and PC-3 cells**. Cells were cultured for 3 days with control (0.1% DMSO), troglitazone (50 and 100 μM; TGZ), 15-deoxy-prostaglandin J_2 _(5.6 and 10 μM; 15dPGJ2) or rosiglitazone (10 μM; RGZ). The effect of these ligands on the proportion of cells in each phase of the cell cycle was determined. Data shown is representative of two experiments.

Given the similar response profiles to the different PPARγ ligands in all bladder and prostate carcinoma cell lines examined, the effect of TGZ, 15dPGJ2 and RGZ on cell cycle regulation was examined in more detail in TSU-Pr1 and PC-3 cell lines (Figure 5). TGZ produced a dose-dependent cell cycle arrest in G_1 _phase both TSU-Pr1 and PC-3 cell lines. In contrast to this 15dPGJ2 dose dependently increased the proportion of subG_1 _cells, accompanied by an arrest in G_2 _phase in TSU-Pr1 and PC-3 cell lines. The effect of 5.6 uM 15dPGJ2 on the proportion of cells in the subG_1 _peak was more marked in PC-3 cells. A G_1 _arrest was observed in response to RGZ treatment in both cell lines. In addition a small increase in the proportion of cells in the subG_1 _peak was observed in response to RGZ treatment in TSU-Pr1 cells.

### TGZ and 15dPGJ2 alter levels of caspase-3/7 activation and DNA fragmentation

**Figure 6:**
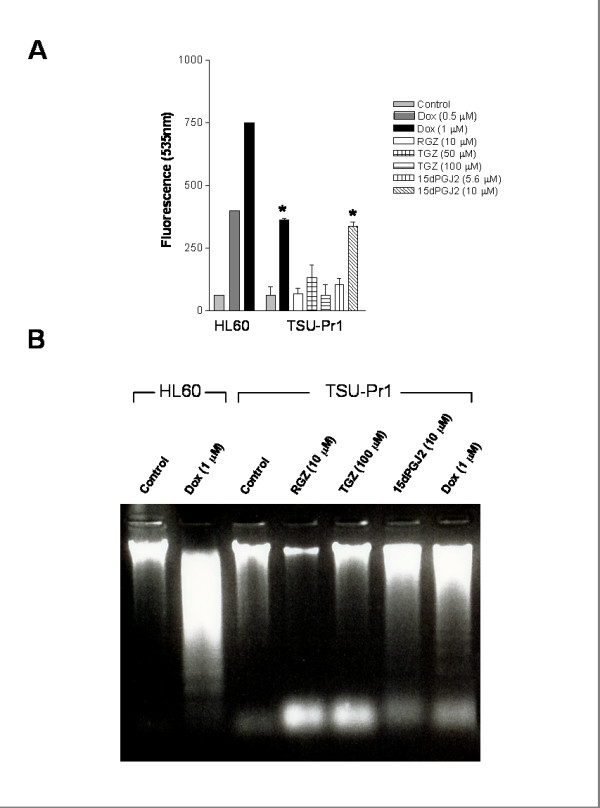
**Effect of PPARγ ligands on apoptosis**. The effect of PPARγ ligands on apoptosis was measured using caspase-3/7 activation (A) and DNA fragmentation (B). A, HL60 and TSU-Pr1 cells were treated with either control (0.1% DMSO), Doxorubicin (0.5 or 1 μM; Dox). TSU-Pr1 cells were also treated with troglitazone (50 and 100 μM; TGZ), 15-deoxy-prostaglandin J_2 _(5.6 and 10 μM; 15dPGJ2) or rosiglitazone (10 μM; RGZ) for 48 hrs. Levels of activated caspase-3/7 were determined by fluorescence measured at 535 nm. Values are mean ± SEM of 2 experiments performed in triplicate. B, HL60 and TSU-Pr1 cells were treated with Doxorubicin (1 μM; Dox), troglitazone (100 μM; TGZ), 15-deoxy-prostaglandin J_2 _(10 μM; 15dPGJ2) or rosiglitazone (10 μM; RGZ) for 72 hrs. DNA was extracted and run on a 1 % agarose gel. Statistical significance * *P *< 0.01, different to TSU-Pr1 control (ANOVA followed by Dunnett post test).

Doxorubicin (Dox) induces apoptosis in the human myeloid leukaemia cell line HL60 [37] and was used as a positive control for both caspase-3/7 activation and DNA fragmentation (Figure 6A and 6B). Induction of apoptosis was investigated by measurement of levels of caspase-3/7 activation after PPARγ ligand treatment in TSU-Pr1 (Figure 6A) and PC-3 (data not shown) cell lines. Both Dox (1 μM) and 15dPGJ2 (10 μM) induced significant caspase-3/7 activation in TSU-Pr1 (Figure 6A) and PC-3 cells, while TGZ (100 μM) induced a small but significant increase in caspase-3/7 activation in PC-3 cells. DNA fragmentation was examined to further investigate the induction of apoptosis. Dox induced DNA fragmentation in the HL60 and TSU-Pr1 cell lines (Figure 6B) after 72 hours of ligand treatment. DNA laddering was induced by 15dPGJ2 (10 μM) treatment in TSU-Pr1 (Figure 6B) and PC-3 (data not shown) cell lines. TGZ (100 μM) did not induce DNA laddering in any sample examined. RGZ (10 μM) did not induce measurable caspase 3/7 activation or DNA laddering in either TSU-Pr1 (Fig 6) or PC-3 (data not shown) cell lines suggesting that the increase in the proportion of cells in the subG_1_peak of RGZ (10 μM) in TSU-Pr1 cells observed in Fig 5 may have been due to cells undergoing necrosis.

## Discussion

Thiazolidinedione ligands have been shown to inhibit the growth of multiple cancer cell lines [2,6,17,38], however it is becoming apparent that these antineoplastic effects cannot always be attributed to PPARγ activation. Reports of PPARγ ligand induced growth inhibition demonstrate three important points: i) Growth inhibition can be specific to members of the thiazolidinedione family [18,19] ii) Growth inhibition can be selective for an endogenous PPARγ ligand compared to synthetic ligand [22], iii) PPARγ ligand mediated growth inhibition is PPARγ-independent in some cancer types [24,39]. Clearly the response to PPARγ ligands and the involvement of PPARγ in these effects differs widely amongst carcinoma types and thus each carcinoma type must be specifically examined. The current study investigated the response to an array of thiazolidinedione agonists and an endogenous PPARγ agonist, 15dPGJ2, in bladder and prostate carcinoma cell lines of differing metastatic potential. In addition we have utilised the selective PPARγ antagonist GW9662 to elucidate the involvement of this receptor in the growth inhibitory effects observed.

We detected profound growth inhibition in response to TGZ and 15dPGJ2 in all bladder and prostate carcinoma cell lines. These results are consistent with the growth inhibitory effects reported by Yoshimura et al. albeit that in our hands 15dPGJ2 generally exerted more potent effects on prostate and bladder cell lines while TGZ was comparatively less potent. The different assays or vehicle utilised may account for these observations. We also found that RGZ and PGZ did not inhibit cell number in the bladder or prostate carcinoma cell lines used in this study. We confirmed that at the dose used in this study, RGZ was capable of inducing adipogenesis in NIH3T3-L1 cells. These findings are similar to the little or no effect on cellular growth exerted by RGZ and PGZ in other cancer systems [18,40]. Our results demonstrate that the known affinities of PPARγ ligands for PPARγ did not predict their relative potency. In other studies using prostate cancer cell lines, RGZ has been shown to inhibit cell number in PC-3 cells [2,34,41]. From our study it is clear that the thiazolidinedione family are not acting through PPARγ. When the mechanism is elucidated it will be important to investigate the activity of the relevant pathway in the various PC-3 cell lines held in different laboratories to determine if this will explain the variation in response to RGZ.

In breast cancer, the PPARγ antagonist GW9662 has recently been used to definitively exclude PPARγ activation in apoptotic and proliferative responses to selected PPARγ agonists [24,39]. At a concentration which inhibits RGZ and TGZ induced adipogenesis in 3T3-L1 cells in our hands, GW9662 did not reverse the growth inhibitory effects of TGZ or 15dPGJ2 in any prostate or bladder cell line examined. Our findings demonstrate that the effects induced by TGZ and 15dPGJ2 in prostate and bladder carcinoma are PPARγ-independent. Whilst these findings are consistent with PPARγ-independent growth inhibition observed in some cancer types, it has been clearly demonstrated in other cases that thiazolidinedione agonists and 15dPGJ2-induced growth inhibition is PPARγ-dependent [21,25,26,42,43]. In further support of a PPARγ-independent mechanism, the TZD ligands and 15dPGJ2 produced comparable growth inhibition in the TSU-Pr1/TSU-Pr1-B1/TSU-Pr1-B2 bladder carcinoma series despite increasing PPARγ expression. Likewise, the levels of PPARγ expression varied significantly amongst the prostate cell lines examined and these differing levels did not associate with the observed growth inhibition.

Further to the PPARγ-independent effects of TGZ and 15dPGJ2 discovered in prostate and bladder carcinoma, our study also demonstrates that TGZ and 15dPGJ2 induce growth inhibition via different mechanisms. TGZ induced cell cycle arrest, whilst 15dPGJ2 treatment resulted in apoptosis. This provides further evidence that these PPARγ ligands implement unique intracellular signalling pathways. Recently, TGZ-induced apoptosis in PC-3 cell lines was shown to involve reduced association of Bcl-2 and Bcl-xL with Bak, leading to caspase-dependent apoptosis [15]. The capability of TGZ to induce apoptosis in this system may be reflective of the larger percentage of control cells in G2/M phase of the cell cycle. Thus, TGZ may be able to inhibit the growth of both actively proliferating tumours and tumours with a lower mitotic rate more typically observed in prostate cancer. The growth arrest and DNA damage-inducible gene 45 (GADD45) has been implicated in TGZ-induced apoptosis in breast carcinoma cell lines [40]. Other potential PPARγ-independent mechanisms that may induce apoptosis include the generation of reactive oxygen species [21] and induction of cellular acidosis [39]. Recently, Chintharlapalli et al. [44] demonstrated that PPARγ agonists induce apoptosis in colon cancer through PPARγ receptor independent actions involving early growth response-1 and NSAID-activated-gene-1.

While treatment with thiazolidinedione ligands has elicited both preventative and deleterious effects in colon and breast cancer [7,45,46], in prostate cancer certain thiazolidinedione ligands exhibit promising antineoplastic properties. TGZ inhibits primary prostatic tumour growth in immunodeficient mice, and in short term culture of human prostate tissue induces selective necrosis of cancer cells while sparing the adjacent normal tissue [2]. In addition, TGZ has been shown to stabilise and reduce PSA levels in clinical trials of patients with advanced prostate cancer [5,28,29]. The potential clinical utility of TGZ reinforces the need to understand the mechanism of action of various thiazolidinedione and other PPARγ ligands. New evidence suggests PPARγ may play a role in the differentiation of epithelia [47,48], which is an exciting prospect in the field of cancer research. Our data indicates RGZ and PGZ would be useful candidates to study the role of up-regulated PPARγ in prostate and bladder carcinoma as these ligands do not exhibit PPARγ-independent growth inhibitory effects. Our current findings demonstrate that 15dPGJ2 and TGZ induce growth arrest in bladder and prostate carcinoma cell lines in a PPARγ-independent manner, and via distinct mechanisms. Further characterisation and elucidation of the molecular mechanisms underlying the antineoplastic effects of TGZ and 15dPGJ2 will be important for clinical development and utilization of these agents.

## Conclusion

PPARγ is overexpressed in multiple cancer types and the role of this receptor in carcinoma progression has been widely studied using the family of high affinity thiazolidinedione PPARγ agonists. However, it has recently been demonstrated that PPARγ ligands can exert PPARγ-independent biological responses including growth arrest and apoptosis in selected carcinoma types. In this paper we demonstrate not only reduced survival in response to certain PPARγ ligands, but that these ligands differentially induce growth arrest or apoptosis in bladder and prostate cell lines in a PPARγ-independent manner. This study highlights the need to elucidate the distinct PPARγ-independent mechanisms of action of individual PPARγ ligands prior to clinical exploration. Our data also suggests that in studying the activation of PPARγ, careful consideration should be given to the ligand employed.

## Competing interests

The authors declare they have no competing interests.

## Authors' contributions

CLC conducted all experiments in this paper and drafted the manuscript. DMT designed and aided in the FACS experiments. CLC, DMT, EWT and EDW provided intellectual input and critically reviewed the manuscript.

## Pre-publication history

The pre-publication history for this paper can be accessed here:
